# Generative-augmented multimodal neuroaffective decoding for product evaluation: toward neurofeedback-driven human-in-the-loop co-design

**DOI:** 10.3389/fnhum.2026.1997654

**Published:** 2026-10-21

**Authors:** Ming Deng

**Affiliations:** Department of Industrial Design, School of Mechanical Engineering and Automation, Northeastern University, Shenyang, China

**Keywords:** electroencephalography, generative artificial intelligence, human-in-the-loop, Kansei engineering, multimodal fusion, neuroadaptive design, neuroaffective computing

## Abstract

**Background:**

Kansei engineering traditionally relies on self-reported evaluations of product impressions, which are susceptible to contextual influences, recall bias, and assessment timing. Multimodal psychophysiological measurements offer complementary insights into affective responses, but integrating heterogeneous signals with limited sample sizes remains challenging. This study proposes Generative-Augmented Multimodal Affective Fusion (GAMAF) to predict perceived attractiveness, innovativeness, and friendliness in product evaluation.

**Methods:**

GAMAF integrates feature-space conditional Wasserstein generative adversarial learning with gradient penalty (cWGAN-GP), cross-modal attention, graph-based interactions, and adaptive modality weighting. Fifty participants evaluated three automobile cockpit designs, yielding 150 participant–stimulus trials after multimodal quality control. Electroencephalography (EEG), eye tracking (ET), and peripheral physiological signals were integrated and evaluated using nested participant-wise cross-validation.

**Results:**

GAMAF achieved mean outer-fold R² values of 0.810, 0.850, and 0.840 for attractiveness, innovativeness, and friendliness, respectively, each with a standard deviation of 0.016. These values exceeded those of the Multimodal Transformer baseline (0.75, 0.78, and 0.76, respectively). Across the three targets, GAMAF reduced aggregate mean absolute error (MAE) and root mean squared error (RMSE) by approximately 29.6% and 29.7%, respectively. Ablation analyses supported the integrated architecture, while adaptive modality weights revealed target-dependent patterns.

**Discussion:**

GAMAF demonstrates the potential of generative-augmented multimodal fusion for decoding Kansei-related product impressions. However, performance comparisons remain descriptive because baseline variability and participant-level uncertainty were not quantified. Further validation on unseen product designs and closed-loop design optimization is needed to establish its generalizability and practical utility.

## 1 Introduction

Product evaluation encompasses both functional performance and the affective impressions that shape preference, acceptance, and user experience. Kansei engineering translates these impressions into design-relevant information through semantic assessment and quantitative modeling (Nagamachi, 2002; Schütte et al., 2004). Recent work connects Kansei evaluation with generative product-form design and physiological measurement, including diffusion-based generation and eye-tracking-assisted assessment (Yang et al., 2023; Zhu et al., 2025). These developments motivate evaluation methods that retain users’ explicit judgments while capturing complementary responses during product perception.

Self-reports directly represent consciously experienced judgments but are influenced by scale wording, response timing, memory, and social context. Physiological measurements can complement these reports by characterizing attention allocation, arousal, and task engagement during product viewing. Different response systems can vary partly independently, and similar physiological changes can accompany different psychological processes (Mauss and Robinson, 2009). Affective computing therefore infers affective states from measurable signals rather than assuming a one-to-one physiological mapping (Calvo and D'Mello, 2010; Picard, 1997). For product design, the relevant question is whether physiological information improves the prediction and interpretation of users’ expressed impressions.

EEG, ET, and peripheral physiology offer partly complementary information about cortical activity, visual attention, and autonomic responses. Integration is challenging because these streams differ in measurement units, dimensionality, noise characteristics, and temporal resolution. Multimodal models must address representation, alignment, redundancy, and fusion while retaining modality-specific information (Baltrusaitis et al., 2019). Previous EEG and eye-movement studies support learned multimodal representations and adaptive feature selection for affect recognition (Fu et al., 2023; Zheng et al., 2019). Evidence for continuous prediction of Kansei-oriented product impressions remains more limited.

Psychophysiological experiments also typically involve relatively few independent participants. Repeated trials provide additional observations for model fitting without creating an equivalent number of independent individuals. Small samples and high-dimensional features can produce unstable generalization estimates, while leakage through preprocessing or participant overlap can inflate performance (Kapoor and Narayanan, 2023; Rosenblatt et al., 2024; Varoquaux, 2018). Generative augmentation may regularize learning by enriching the training distribution, but its usefulness depends on the signal, task, and validation design. Research on EEG augmentation, diffusion-based generation, and reconstruction-based domain generalization illustrates both its potential and its task dependence (Rommel et al., 2022; Wang et al., 2024; Zhao et al., 2024).

This study evaluates GAMAF for predicting attractiveness, innovativeness, and friendliness from multimodal psychophysiological features recorded during automobile cockpit evaluation. The framework combines conditional feature-space augmentation, cross-modal attention, graph-based interaction, and adaptive modality weighting. Its contribution is the integration and evaluation of these components within a Kansei-oriented product-evaluation pipeline. Three research questions guide the analysis. RQ1 asks whether GAMAF improves prediction relative to representative early, late, tensor, low-rank, and transformer fusion models. RQ2 examines the predictive contributions of individual architectural components. RQ3 examines whether learned modality weights differ across the three product-impression targets. The evaluation is restricted to the present laboratory task, and latent weights are interpreted as computational diagnostics rather than causal physiological pathways.

## 2 Related work

### 2.1 Kansei evaluation and complementary physiological evidence

Kansei engineering translates subjective product impressions into information for design development and evaluation (Nagamachi, 2002; Schütte et al., 2004). Semantic descriptors require a clear interpretation within the focal product domain and target population. Here, attractiveness, innovativeness, and friendliness represent related but distinct product impressions rather than direct proxies for generic valence or arousal. A visually novel design can appear innovative without being liked, whereas an approachable design need not elicit uniformly high arousal. Appraisal theory links emotional responses to personal meaning but does not establish an invariant sequence from visual perception to cortical appraisal and autonomic activation (Lazarus, 1991).

The three dimensions capture distinct aspects of the cockpit as a visually encountered product environment. Attractiveness concerns overall visual appeal and the perceived coherence of proportions, color, and composition. Innovativeness concerns departure from familiar cockpit configurations and the perceived novelty of interface layouts and product-form concepts. Friendliness concerns the apparent approachability of an environment in which users anticipate interacting with controls and displays. Together, these dimensions distinguish liking, perceived novelty, and approachability without assuming that they vary together. They provide a conceptual basis for this visual evaluation task but do not exhaust cockpit experience or establish usability, driving safety, or real-world acceptance.

Computational Kansei research combines generative product-form synthesis with multimethod evaluation, including eye tracking (Yang et al., 2023; Zhu et al., 2025). GAMAF addresses the complementary question of whether concurrent physiological and behavioral measurements predict observed Kansei ratings. Because self-reports remain the supervised targets, prediction performance establishes correspondence with those ratings rather than an elimination of subjectivity. The framework therefore preserves explicit judgments while evaluating the predictive value of multimodal psychophysiological evidence.

### 2.2 Physiological features and limits of interpretation

EEG provides time-sensitive measures of cortical activity during product viewing, including event-related potentials (ERPs) and spectral features. Frontal alpha asymmetry (FAA) is often discussed in relation to motivational direction, but its sign and interpretation depend on reference choice, preprocessing, channel selection, and the mathematical convention used to compute asymmetry (Smith et al., 2017). The late positive potential (LPP) is sensitive to sustained processing of motivationally relevant stimuli and can be modulated by reappraisal (Hajcak and Nieuwenhuis, 2006). These components are therefore useful candidate predictors, but neither FAA nor LPP is uniquely diagnostic of innovativeness, friendliness, or attractiveness. Their value must be demonstrated within the specific product-evaluation task.

ET complements EEG by characterizing where and how visual attention is allocated. Fixation duration, fixation count, saccade amplitude, transitions between areas of interest (AOIs), and pupil responses can capture different aspects of visual exploration. Pupil diameter is particularly sensitive to cognitive and autonomic influences, but it is also affected by luminance, gaze position, blinks, and display properties (Mathôt, 2018). Consequently, valid pupillometry requires explicit control of the visual environment, careful blink interpolation, baseline correction, and transparent validity criteria (Mathôt and Vilotijević, 2023; Steinhauer et al., 2022). These issues are especially relevant for cockpit renderings that differ in color, spatial layout, and interface complexity.

Peripheral physiology provides a further source of complementary information. Electrodermal activity (EDA) reflects sympathetic sudomotor activity and is commonly decomposed into tonic skin conductance level (SCL) and phasic skin conductance responses (SCRs), which require distinct operational definitions and detection procedures (Benedek and Kaernbach, 2010; Boucsein et al., 2012). Electrocardiography (ECG) provides beat-to-beat intervals from which heart-rate variability (HRV) features can be estimated, although their interpretation depends on recording duration, artifact correction, respiration, and metric choice (Laborde et al., 2017; Shaffer and Ginsberg, 2017; Tarvainen et al., 2014). In particular, the LF/HF ratio should not be interpreted as a direct measure of sympathovagal balance (Billman, 2013). Accordingly, the present study treats peripheral measures as predictive features with bounded physiological interpretation.

Public affective-computing resources such as DEAP and DREAMER have facilitated research using EEG and peripheral physiology, while EmotionMeter demonstrated multimodal fusion of EEG and eye-movement information (Katsigiannis and Ramzan, 2018; Koelstra et al., 2012; Zheng et al., 2019). These datasets provide important methodological precedents, but their stimuli, label definitions, and acquisition protocols differ materially from the present automobile-cockpit evaluation task. Performance reported on categorical emotion-recognition benchmarks therefore cannot be compared directly with regression performance on five-point product-impression ratings. This domain difference motivates a dedicated evaluation framework for Kansei-oriented product assessment.

### 2.3 Generative augmentation and cross-participant generalization

Conditional generative adversarial networks generate samples conditional on observed labels, while Wasserstein training with gradient penalty provides a stable critic objective and a practical approach to enforcing the Lipschitz constraint (Gulrajani et al., 2017; Mirza and Osindero, 2014). Applied to a joint psychophysiological feature vector, conditional generation can enrich underrepresented regions of the training distribution and potentially preserve cross-feature dependencies. However, feature-space generation does not reconstruct raw EEG waveforms, gaze trajectories, or cardiac cycles and therefore cannot, by itself, establish biological realism or preservation of temporal phase relationships. Synthetic samples must instead be evaluated for distributional coverage, label consistency, memorization risk, and downstream usefulness on held-out real observations.

Recent studies have addressed limited-data and cross-participant variability through several complementary mechanisms. Diffusion-based augmentation has been used to generate additional EEG training signals, DMMR combines reconstruction and augmentation to support cross-subject domain generalization, and graph-based adversarial models explicitly target cross-subject distribution shifts (Chen et al., 2024; Wang et al., 2024; Zhao et al., 2024). Cross-dataset domain adaptation further illustrates the extent to which participants, devices, and stimulus sets can alter EEG distributions (Imtiaz and Khan, 2025). These approaches motivate the present emphasis on participant-grouped validation and provide useful directions for future extensions, although they were not included as direct baselines in the present experiment.

Augmentation quality depends on the validation boundary. A participant-wise split alone does not prevent leakage if preprocessing, feature selection, generator fitting, augmentation settings, or hyperparameters incorporate held-out observations. Data-dependent operations must remain within the training partition, with model selection separated from outer-fold performance estimation (Kapoor and Narayanan, 2023; Kapoor et al., 2024; Varma and Simon, 2006). Synthetic observations may enrich the training distribution but do not increase the number of independent participants available for inference.

### 2.4 Attention, graph interaction, and adaptive fusion

Multimodal fusion methods differ principally in how they represent interactions among modality-specific features. Tensor Fusion Networks (TFN) model explicit higher-order interactions through tensor products, low-rank multimodal fusion (LMF) reduces the computational burden of this representation, and the Multimodal Transformer (MulT) uses cross-modal attention to exchange information between modality streams (Liu et al., 2018; Tsai et al., 2019; Zadeh et al., 2017). Early feature concatenation can also capture nonlinear relationships when coupled with a nonlinear predictor, while late fusion aggregates modality-specific decisions. Accordingly, the relevant empirical question is not whether one family is intrinsically superior, but whether the proposed combination of interaction modeling and adaptive weighting provides reproducible gains for the present product-evaluation task.

Graph attention networks learn weighted neighborhood aggregation and can represent structured dependencies among learned features (Veličković et al., 2018). EEG emotion-recognition research has used graph neural networks to model relations among channels, although GAMAF adopts a different abstraction in which each node represents an entire modality rather than an anatomical sensor (Zhong et al., 2022). The original graph-attention formulation also has known limitations in query-dependent neighbor ranking, motivating later variants such as GATv2 (Brody et al., 2022). In GAMAF, graph coefficients and gating weights are therefore interpreted as learned computational weights rather than direct measures of physiological connectivity or causal importance. This distinction is consistent with broader evidence that attention coefficients alone do not constitute a complete explanation of model predictions (Jain and Wallace, 2019; Serrano and Smith, 2019).

## 3 Materials and methods

### 3.1 Participants, stimuli, and rating task

Fifty healthy adults (25 males and 25 females) participated in the experiment, and each participant evaluated three automobile cockpit renderings, yielding 150 participant–stimulus trials. All 150 trials satisfied the prespecified multimodal quality-control criteria and were retained for subsequent statistical and predictive analyses. Participants were 20–30 years old and reported normal or corrected-to-normal vision. An *a priori* power analysis was conducted in G*Power. Assuming a medium effect size (*f* = 0.25), *α* = 0.05, and statistical power of 0.95, the minimum required sample size was 24; the recruited sample therefore exceeded this requirement (Lakens, 2022). The study protocol was reviewed and approved by the Ethics Committee of Northeastern University (Approval No. NEU-EC-2023B011S; 13 March 2023).

The stimuli comprised three high-resolution renderings of automobile cockpit designs that differed in proportion, color treatment, and interface complexity. Display and viewing conditions were identical across stimuli. Participants sat approximately 60 cm from the screen in a sound-attenuated laboratory maintained at 22 °C. Each trial included a 500-ms fixation period, a 6,000-ms viewing interval, and self-paced ratings of attractiveness, innovativeness, and friendliness. The five-point semantic-differential scales were anchored by unattractive–attractive, conventional–innovative, and unfriendly–friendly, respectively. Regression treated the numerical coding of these ordered categories as approximately interval-scaled, which limits the interpretation of small predicted differences.

### 3.2 Acquisition, preprocessing, and feature definitions

EEG, ET, EDA, and ECG were recorded synchronously during product viewing to capture complementary neural, visual-attentional, and autonomic responses. EEG was acquired with a Brain Products actiCHamp system using 64 active electrodes placed according to the international 10–20 system, referenced online to Cz and grounded at AFz. Electrode impedances were maintained below 5 kΩ and signals were digitized at 1,000 Hz. Gaze was recorded with a Tobii Pro Spectrum at 600 Hz after a nine-point calibration, with mean calibration error constrained to less than 0.4° of visual angle. Peripheral signals were recorded using a Biopac MP160: EDA electrodes were attached to the volar surfaces of the distal phalanges of the index and middle fingers of the non-dominant hand, and ECG was obtained using a standard bipolar Lead II configuration. Lab Streaming Layer (LSL), controlled through OpenViBE 3.5.0, was used to synchronize acquisition across devices.

LF power, HF power, and LF/HF were excluded from the six-second trial-level feature matrix and all final predictive analyses. No extended-window spectral HRV substitute was used in GAMAF or the baseline models. A single cycle at the lower LF boundary of 0.04 Hz lasts 25 s, exceeding the viewing interval. A high ECG sampling rate cannot compensate for insufficient observation duration. ECG-derived predictors were therefore restricted to RMSSD and SDNN. These ultra-short-window descriptors depend on valid-beat counts and artifact correction and are not interchangeable with conventional longer-window HRV measures (Laborde et al., 2017; Shaffer and Ginsberg, 2017).

### 3.3 Prediction task and notation

Let i index participants, j index cockpit stimuli, and k index the three rating targets. Each participant–stimulus trial is represented by modality-specific feature vectors for EEG, ET, and peripheral physiology, with the peripheral vector combining EDA- and ECG-derived features. The corresponding outcome is the observed five-point rating for target k. The final analysis dataset comprises *N* = 50 participants and J = 3 stimuli, giving T = NJ = 150 participant–stimulus trials. All 150 trials were retained after multimodal quality control, and each trial contributes observations for all three rating targets. As defined in Equation 1, the model maps the three modality-specific vectors to one predicted rating, and a separate target-specific prediction head is trained for attractiveness, innovativeness, and friendliness.

$${\hat{y}}_{\mathrm{ijk}}=f_{\psi_{k}}(x_{\mathrm{ij}}^{\left(\mathrm{EEG}\right)},x_{\mathrm{ij}}^{\left(\mathrm{ET}\right)},x_{\mathrm{ij}}^{\left(\mathrm{PER}\right)})$$ (1)

### 3.4 Participant-wise validation and preprocessing boundary

Generalization was evaluated using five outer cross-validation folds grouped by participant, with each individual’s trials assigned to the same fold. Each split comprised 40 outer training participants and 10 held-out participants, corresponding to 120 training and 30 test trials. Data-dependent preprocessing was fitted within the training partition and applied to held-out observations using the fitted parameters. Equation 2 defines standardization for feature q in modality m and fold ℓ using the training-partition mean and standard deviation. Constant features were removed within the training fold rather than divided by zero.

$${\bar{x}}_{\mathrm{ijq}}^{\left(m\right)}=\frac{x_{\mathrm{ijq}}^{\left(m\right)}-\mu_{\mathrm{mq}}^{\left(\ell ,\text{train}\right)}}{s_{\mathrm{mq}}^{\left(\ell ,\text{train}\right)}}$$ (2)

Hyperparameters, feature-selection thresholds, augmentation ratios, and stopping decisions were selected through participant-grouped inner validation within each outer training fold. For predictor early stopping, 20% of the outer training participants formed the validation subset, with all trials from an individual retained together. Each outer training partition therefore comprised 32 fitting participants and eight validation participants. The selected pipeline was evaluated once on the corresponding real outer test fold. This nested design separates model selection from performance estimation and prevents test-participant information from entering preprocessing, augmentation, or tuning (Kapoor et al., 2024; Varma and Simon, 2006).

### 3.5 Conditional generative augmentation

GAMAF performs feature-space augmentation using a conditional Wasserstein generative adversarial network with gradient penalty (cWGAN-GP). A Wasserstein objective is used instead of the binary cross-entropy objective of a conventional conditional GAN because it provides a smoother critic signal and improves training stability. For one target, the generator Gθ maps a random noise vector z and a conditioning rating y to a synthetic joint multimodal feature vector x̃, as expressed in Equation 3. The noise vector is sampled from a standard multivariate normal distribution, while the critic Dφ returns an unconstrained real-valued score rather than a calibrated probability of whether a sample is real (Gulrajani et al., 2017; Mirza and Osindero, 2014).

$$\tilde{x}=G_{\theta }(z,y),z∼N(0,I_{d_{z}})$$ (3)

The critic and generator objectives are given in Equations 4, 5, respectively. The gradient penalty in Equation 6 is computed at interpolated points x̂ between real and generated samples that share the same conditioning value. The penalty coefficient was set to λGP = 10, consistent with the standard WGAN-GP formulation. This regularization discourages excessively steep critic gradients and stabilizes training without requiring weight clipping.

$${ℒ}_{D}=E_{z,y}[D_{\varphi }(G_{\theta }(z,y),y)]-E_{x,y}[D_{\varphi }(x,y)]+\lambda_{\mathrm{GP}}{ℒ}_{\mathrm{GP}}$$

$$L_{\mathrm{GP}}=E_{\hat{x},y}[{\left({\left\|\nabla_{\hat{x}}D_{\varphi }(\hat{x},y)\right\|}_{2}-1\right)}^{2}]$$ (4)

$${ℒ}_{G}=-E_{z,y}[D_{\varphi }(G_{\theta }(z,y),y)]$$ (5)

$$\hat{x}=\mathrm{\varepsilon x}+(1-\varepsilon )\tilde{x},\varepsilon ∼U(0,1)$$ (6)

Synthetic observations were generated jointly across all retained multimodal features so that the generator could learn dependencies spanning EEG, ET, and peripheral physiology. Conditioning labels were sampled exclusively from the training data, and no held-out rating was used during generation, feature selection, or predictor fitting. After generation, the joint synthetic vector was partitioned back into modality-specific inputs before entering the corresponding encoders. Because augmentation was performed in feature space, the procedure enriches the statistical training distribution but does not synthesize raw EEG, gaze trajectories, EDA waveforms, or ECG signals and therefore should not be interpreted as reproducing their fine-grained temporal dynamics.

The augmentation ratio ρ, defined in Equation 7, was defined relative to the number of real observations in each training partition. Thus, ρ = 2 corresponds to 200% augmentation: for every n real training observations, 2n synthetic observations are generated, yielding 3n total samples for model fitting. Augmentation ratios were evaluated within the training data so that the selected ratio reflected downstream performance rather than distributional similarity alone.

$$\rho =\frac{T_{\text{synthetic}}}{T_{\text{real},\text{train}}},T_{\text{augmented}}=(1+\rho )T_{\text{real},\text{train}}$$ (7)

### 3.6 Feature selection

Feature selection was designed to retain informative but non-redundant psychophysiological predictors while limiting the dimensionality of the small-sample learning problem (Breiman, 2001; Chen and Guestrin, 2016; Tibshirani, 1996). Random Forest and XGBoost models first provided nonlinear importance rankings. Pairwise redundancy was then assessed using normalized mutual information (NMI), estimated directly for continuous features with the non-parametric Kraskov–Stögbauer–Grassberger k-nearest-neighbor estimator (*k* = 3). For feature pairs with NMI > 0.75, the feature with the lower XGBoost importance was removed. Finally, LASSO regression imposed an L1 penalty on the remaining predictors to obtain a sparse feature subset. The regularization parameter λ was tuned by five-fold inner cross-validation using only the outer training partition. The complete feature-selection sequence—including NMI estimation, tie resolution, LASSO tuning, and cWGAN-GP fitting—was repeated independently within every outer fold to prevent leakage from held-out participants.

### 3.7 Cross-modal attention, graph interaction, and adaptive weighting

Each modality-specific encoder projected its retained feature vector into a shared dₕ = 64 representation using a learned affine transformation followed by LeakyReLU activation. In Equation 8, dₘ denotes the number of retained input features for modality m. Mapping all modalities to a common dimensionality facilitates subsequent cross-modal interaction while preserving separate encoder parameters for EEG, ET, and peripheral physiology.

$$h_{m}=\text{LeakyReLU}(W_{m}{\bar{x}}^{\left(m\right)}+b_{m}),W_{m}\in {\mathbb{R}}^{64\times d_{m}}$$ (8)

Cross-modal interaction was implemented with scaled dot-product attention following Vaswani et al. (2017). For each attention head r, the representation of one modality served as the query, while the two remaining modalities jointly formed the key-value sequence. This construction yields a nontrivial attention distribution over the two complementary modalities and avoids the degeneracy of a singleton-key softmax. Equations 9–11 define the query-key similarity, normalized attention coefficients, and residual-normalized output for each modality.

$$S_{-m}=[h_{n}:n\neq m]\in {\mathbb{R}}^{2}\times^{64}$$ (9)

$$c_{m}^{\left(r\right)}=\text{softmax}(\frac{q_{m}^{\left(r\right)}K_{-m}^{(r)T}}{\sqrt{d_{a}}})V_{-m}^{\left(r\right)}$$ (10)

$${\hat{h}}_{m}=\text{LayerNorm}(h_{m}+W_{0}{\left[{c_{m}}^{\left(1\right)}∥\cdots ∥{c_{m}}^{\left(4\right)}\right]}^{T})$$ (11)

Each attention head used a single query row and two key/value rows representing the complementary modalities. Softmax operated across the key-token dimension. Outputs from four heads were concatenated, projected, combined with a residual connection, and layer-normalized. This operation aligns trial-level representations in a common feature space. It does not estimate millisecond-scale temporal alignment between EEG samples and gaze events because the inputs are extracted features rather than raw sequences.

The cross-attended representations were subsequently treated as three nodes in a fully connected computational graph with self-connections. A graph attention layer transformed each node and aggregated information from all neighboring modality nodes (Veličković et al., 2018). In Equations 12–14, the graph projection and attention vector parameterize the edge score, normalized coefficient, and updated node state. This graph is an architectural representation of multimodal dependency rather than an anatomical connectivity graph.

$$e_{\mathrm{mn}}=\text{LeakyReLU}(a^{T}[W_{g}{\hat{h}}_{m}\|W_{g}{\hat{h}}_{n}])$$ (12)

$$\alpha_{\mathrm{mn}}=\frac{\exp(e_{\mathrm{mn}})}{\sum_{j\in N_{m}}\exp(e_{\mathrm{mj}})}$$ (13)

$$h_{m}^{\prime }=\mathrm{ELU}(\sum_{n\in N_{m}}\alpha_{\mathrm{mn}}W_{g}{\hat{h}}_{n})$$ (14)

Directional attention permits the coefficient from modality m to modality n to differ from the reverse coefficient. These coefficients represent computational message weighting rather than physical coupling. GAMAF uses the original GAT scoring rule. Query-dependent alternatives such as GATv2 differ in their neighbor-ranking behavior and constitute distinct architectures requiring separate evaluation (Brody et al., 2022).

A gating network then assigned target-dependent normalized weights to the graph-updated modality representations. The gate score is a learned scalar that reflects the model’s use of the available representations for a given prediction; it is not a calibrated measure of physiological information density. Equations 15–17 define the gate, the fused representation, and the scalar regression head, with index k indicating that the gating behavior can differ across attractiveness, innovativeness, and friendliness.

$$g_{\mathrm{mk}}=u_{\mathrm{mk}}^{T}\tanh(W_{\mathrm{ak}}h_{m}^{\prime }+b_{\mathrm{ak}})$$ (15)

$$w_{\mathrm{mk}}=\frac{\exp(g_{\mathrm{mk}})}{\sum_{n\in ℳ}\exp(g_{\mathrm{nk}})}$$ (16)

$$Z_{k}=\sum_{m\in ℳ}w_{\mathrm{mk}}h_{m}^{\prime },{\hat{y}}_{k}=r_{\omega_{k}}(Z_{k})$$ (17)

For finite logits, the gate satisfies 0 < wₘₖ < 1 and Σₘwₘₖ = 1. Input- and target-dependent weights allow different modality representations to be emphasized for different product impressions. Because graph propagation has already mixed cross-modal information, a large EEG-node weight does not quantify the exclusive contribution of EEG sensors. Gate weights are therefore interpreted comparatively at the representation level.

The regression head comprised a 32-unit hidden layer with ELU activation followed by a single linear output. The fused representation entering this head was 64-dimensional. Model training minimized squared prediction error defined in Equation 18, while optimization and stopping hyperparameters were selected within the inner participant-grouped validation procedure described in Section 3.4. This separation ensured that tuning decisions did not use the outer held-out participants.

$${ℒ}_{\mathrm{reg},k}=\frac{1}{T_{\text{augmented}}}\sum_{t=1}^{T_{\text{augmented}}}{\left(y_{\mathrm{tk}}-{\hat{y}}_{\mathrm{tk}}\right)}^{2}$$ (18)

### 3.8 Evaluation metrics and statistical analysis

Predictive performance was assessed using mean absolute error (MAE), root mean squared error (RMSE), and the coefficient of determination (*R*^2^). Equations 19–21 define these metrics for real held-out observations. MAE and RMSE are expressed in five-point rating units and quantify prediction-error magnitude. *R*^2^ measures performance relative to the held-out target mean, can be negative on unseen data, and is distinct from classification accuracy.

$${\mathrm{MAE}}_{k}=\frac{1}{T_{\text{test}}}\sum_{t=1}^{T_{\text{test}}}|y_{\mathrm{tk}}-{\hat{y}}_{\mathrm{tk}}|$$ (19)

$${\text{RMSE}}_{k}=\sqrt{\frac{1}{T_{\text{test}}}\sum_{t=1}^{T_{\text{test}}}{\left(y_{\mathrm{tk}}-{\hat{y}}_{\mathrm{tk}}\right)}^{2}}$$ (20)

$$R_{k}^{2}=1-\frac{\sum_{t=1}^{T_{\text{test}}}{\left(y_{\mathrm{tk}}-{\hat{y}}_{\mathrm{tk}}\right)}^{2}}{\sum_{t=1}^{T_{\text{test}}}{\left(y_{\mathrm{tk}}-{\bar{y}}_{\text{test},k}\right)}^{2}}$$ (21)

Performance summaries were obtained from real predictions in participant-grouped outer test folds. Section 4.6 reports GAMAF fold-level *R*^2^ values, arithmetic means, and sample standard deviations using K − 1 as the denominator for K = 5 folds. Fold dispersion describes variation across participant splits rather than confidence intervals or independent random-seed repetitions. Comparative tables present aggregate R^2^, MAE, and RMSE estimates. Fold-level error dispersion, corresponding baseline fold statistics, and repeated-seed uncertainty were not quantified. Pooled out-of-fold metrics and arithmetic means of fold metrics are not generally interchangeable. Participant-level bootstrap uncertainty was not estimated, and any subsequent resampling analysis should retain each participant’s trials together.

Synthetic-feature quality was characterized using maximum mean discrepancy (MMD), Wasserstein distance, and an auxiliary downstream evaluation. MMD measures distributional discrepancy in a kernel-induced space, whereas Wasserstein distance quantifies the cost of transporting probability mass between distributions (Gretton et al., 2012). These measures were used together because a single similarity index cannot establish label fidelity, diversity, or predictive utility. The auxiliary accuracy and F1 score were therefore interpreted as downstream utility measures on real evaluation data rather than as stand-alone evidence of synthetic-data realism (Table 1).

**Table 1:** Physiological feature definitions and interpretive boundaries.

Signal	Feature	Operational definition	Interpretive boundary
EEG	P100 amplitude	Peak at 80–120 ms; O1, O2, Oz (μV)	Early visual response; amplitude depends on peak selection.
EEG	N200 amplitude	Negative peak at 180–250 ms; Fz, Cz, Pz (μV)	Task-dependent ERP; not specific to novelty.
EEG	P300 amplitude	Positive peak at 250–400 ms; Cz, Pz (μV)	Task-related processing; amplitude depends on channel aggregation.
EEG	LPP amplitude	Mean at 400–800 ms; Pz, POz (μV)	Sustained ERP; not a direct friendliness measure.
EEG	Theta power	4–7 Hz; Fz, FCz	Power depends on spectral estimation and normalization.
EEG	Alpha power	8–13 Hz; parietal/occipital sites	Power depends on electrode selection, reference, and scaling.
EEG	Beta power	14–30 Hz; C3, C4	Spectral descriptor; not specific to concentration.
EEG	Gamma power	31–50 Hz	High-frequency power is sensitive to muscle artifacts.
EEG	FAA	ln Pα(F4) − ln Pα(F3); dimensionless	Positive values denote greater right alpha power.
ET	Mean fixation duration	Σ fixation durations / fixation count (ms)	Dependent on fixation detection and minimum-duration thresholds.
ET	Fixation count	Number during the 6-s viewing window	Viewing behavior; not equivalent to search efficiency.
ET	Mean saccade amplitude	Mean angular displacement (degrees)	Dependent on saccade detection and valid-sample criteria.
ET	Mean pupil change	Mean stimulus diameter minus baseline (mm)	Sensitive to luminance, gaze position, and blink artifacts.
ET	Peak pupil change	Task-evoked peak at 1–3 s (mm)	Baseline- and extraction-dependent; not specific to pleasure.
ET	AOI fixation-time ratio	Fixation time in target AOI / 6 s	AOI-specific viewing measure; affected by invalid gaze samples.
ET	Transition entropy	−Σₐᵦ pₐᵦ log₂ pₐᵦ (bits)	Joint AOI transition probabilities; 0 log₂ 0 = 0.
EDA	SCL	Mean tonic component (μS)	Tonic conductance depends on baseline and decomposition.
EDA	Summed SCR amplitude	Sum of phasic peaks > 0.01 μS	Summed response amplitude, distinct from mean or peak amplitude.
EDA	SCR frequency	Number of responses / window duration in minutes	Dependent on observation duration and overlapping responses.
ECG	Mean NN interval	Mean artifact-corrected normal-to-normal interval (ms)	Candidate descriptor; excluded from the final ECG predictor set.
ECG	SDNN	Sample SD of normal-to-normal intervals (ms)	Retained time-domain descriptor; duration- and valid-beat-count dependent.
ECG	RMSSD	Root mean square of successive NN differences (ms)	Retained time-domain descriptor; sensitive to short-window reliability.
ECG	LF power	Power at 0.04–0.15 Hz (ms^2^)	Excluded from the trial-level matrix and all final predictive analyses.
ECG	HF power	Power at 0.15–0.40 Hz (ms^2^)	Excluded from the trial-level matrix and all final predictive analyses.
ECG	LF/HF ratio	LF divided by HF; dimensionless	Excluded from all final predictive analyses; not a direct measure of sympathovagal balance.

Features are interpreted as task-related descriptors rather than specific physiological markers of individual Kansei constructs. RMSSD and SDNN were the only ECG-derived predictors retained in the trial-level feature matrix. Mean NN interval, LF power, HF power, and LF/HF are listed as excluded candidates. Further feature selection can produce different retained subsets across targets and folds. EEG, electroencephalography; ET, eye tracking; EDA, electrodermal activity; ECG, electrocardiography; NN, normal-to-normal beat interval; FAA, frontal alpha asymmetry; LPP, late positive potential. Interpretive guidance follows Boucsein et al. (2012), Laborde et al. (2017), Smith et al. (2017), and Steinhauer et al. (2022).

Selected physiological variables were summarized across low, medium, and high rating categories using one-way ANOVA and Tukey *post hoc* contrasts. Repeated observations within participants violate the independence assumption of these tests. The statistics are therefore presented as descriptive summaries of category separation rather than confirmatory population-level evidence. Confirmatory inference would require a model accounting for participant dependence and the shared stimuli, such as a suitable mixed-effects model (Bates et al., 2015).

### 3.9 Implementation settings and reproducibility

Table 2 summarizes the implementation settings. The GAMAF predictor used AdamW with β₁ = 0.9, β₂ = 0.999, and weight decay of 10^−4^. The initial learning rate was 10^−3^, with cosine annealing to a minimum of 10^−5^. Training used batch size 32 and a maximum of 200 epochs. Early stopping monitored validation mean squared error (MSE), while R^2^ provided an additional performance diagnostic. Training stopped after 20 consecutive epochs without improvement in validation MSE, and the lowest-MSE checkpoint was restored. Checkpoint selection used the participant-grouped inner validation subset described in Section 3.4 and excluded the outer test fold.

**Table 2:** GAMAF architecture and training hyperparameters.

Component or setting	Implemented value
Validation	Five participant-grouped outer folds; participant-grouped internal validation using 20% of outer training participants; five-fold inner LASSO tuning
Modality encoders	Three separate affine projections; shared representation dimension 64; LeakyReLU activation
Cross-modal attention	Four heads; one query modality and two key/value modalities; output projection, residual connection, and layer normalization
Graph and fusion	Three fully connected modality nodes with self-connections; GAT interaction; normalized target-dependent gating
Regression head	64-dimensional fused input; 32-unit ELU hidden layer; one linear output; squared-error loss
Predictor optimizer	AdamW; β₁ = 0.9, β₂ = 0.999; weight decay 10^−4^
Predictor learning rate	Initial rate 10^−3^; cosine annealing; minimum rate 10^−5^
Predictor training and stopping	Batch size 32; maximum 200 epochs; validation MSE with additional R^2^ monitoring; patience 20 epochs; restore lowest-MSE checkpoint
cWGAN-GP optimizer	Adam; β₁ = 0.5, β₂ = 0.9; fixed learning rate 2 × 10^−4^
cWGAN-GP training	Batch size 32; maximum 500 epochs; critic-to-generator update ratio 5:1; gradient-penalty coefficient 10; standard normal input noise
Augmentation and selection	200% synthetic-to-real augmentation ratio; KSG neighbor count 3; NMI threshold 0.75; inner-tuned LASSO penalty

Internal validation used 20% of outer training participants, with all trials from each individual retained together. Retained input dimensions can differ across targets and folds. Generator fitting and predictor optimization remained within the participant-grouped training and model-selection boundaries.

The cWGAN-GP generator and critic used Adam with β₁ = 0.5, β₂ = 0.9, learning rate 2 × 10^−4^, and batch size 32. Adversarial training comprised five critic updates per generator update and a maximum of 500 epochs, with gradient-penalty coefficient 10. Generator fitting and feature generation remained within the relevant training partition, excluding outer test samples and labels. The predictor’s validation-MSE stopping rule was specific to supervised regression rather than adversarial optimization.

## 4 Results

### 4.1 Analysis population and synthetic-feature quality

Table 3 summarizes synthetic-feature similarity and auxiliary predictive utility across augmentation ratios. From 50 to 200% augmentation, MMD decreased from 0.045 to 0.012 and Wasserstein distance decreased from 0.089 to 0.024. Auxiliary F₁ increased from 0.695 to 0.808, compared with 0.632 for the non-augmented baseline. At 300 and 400%, distributional distances increased and auxiliary performance declined. Among the tested settings, 200% augmentation showed the most favorable combination of distributional agreement and predictive utility.

**Table 3:** Augmentation-ratio analysis and auxiliary evaluation summary.

Aug. (%)	Original n	Synthetic n	Total n	MMD	WD	Aux. acc. (%)	Aux. F₁
0	150	0	150	N/A	N/A	65.4%	0.632
50	150	75	225	0.045	0.089	71.2%	0.695
100	150	150	300	0.023	0.046	76.8%	0.751
150	150	225	375	0.015	0.031	79.5%	0.782
200	150	300	450	0.012	0.024	81.9%	0.808
300	150	450	600	0.038	0.068	78.1%	0.765
400	150	600	750	0.062	0.112	74.3%	0.720

Sample counts express augmentation ratios relative to the 150 real trials in the full dataset; actual training-partition sizes follow Section 3.4. Synthetic features used for predictive fitting were generated only within training partitions. MMD and Wasserstein distance describe real–synthetic distributional discrepancy, while auxiliary accuracy and F₁ describe downstream utility. Auxiliary evaluation is not a strict train-on-synthetic/test-on-real protocol and does not independently establish biological realism.

### 4.2 Physiological feature summaries

Table 4 summarizes category-level differences for four representative physiological features. The largest descriptive separation was observed for SCR amplitude, *F* (2, 147) = 126.12, *p* < 0.001, *η*^2^ = 0.6318, followed by FAA, *F* (2, 147) = 54.50, *p* < 0.001, *η*^2^ = 0.4258, and pupil diameter, *F* (2, 147) = 51.63, *p* < 0.001, *η*^2^ = 0.4126. LPP amplitude showed a smaller but still notable category effect, *F* (2, 147) = 11.12, *p* < 0.001, *η*^2^ = 0.1314. Taken descriptively, these effect sizes suggest that the selected visual-attentional, cortical, and autonomic features each contain information related to the rating categories, with the strongest separation observed for SCR amplitude.

**Table 4:** Descriptive ANOVA summary across rating categories.

Feature	Source	SS	df	MS	*F*	*p*	*η* ^2^
Pupil diameter (mm)	Between (attractiveness)	10.4915	2	5.2457	51.63	< 0.001	0.4126
	Residual	14.9360	147	0.1016			
	Total	25.4275	149				
FAA	Between (innovativeness)	1.2676	2	0.6338	54.50	< 0.001	0.4258
	Residual	1.7095	147	0.0116			
	Total	2.9771	149				
SCR amplitude (μS)	Between (friendliness)	13.5408	2	6.7704	126.12	< 0.001	0.6318
	Residual	7.8910	147	0.0537			
	Total	21.4318	149				
LPP amplitude (μV)	Between (friendliness)	17.6471	2	8.8236	11.12	< 0.001	0.1314
	Residual	116.6925	147	0.7938			
	Total	134.3396	149				

The one-way ANOVA values describe category-level separation. Because each participant contributed repeated trials, the observations are not fully independent; these statistics are therefore interpreted descriptively rather than as the sole basis for population-level inference. η^2^ = SSbetween/SStotal. SS, sum of squares; MS, mean square.

Table 5 reports descriptive Tukey contrasts between rating categories. All three contrasts for pupil diameter, FAA, and SCR amplitude had adjusted *p* < 0.001 and large standardized differences. For LPP amplitude, the low–medium and low–high contrasts had adjusted *p* < 0.001. The medium–high contrast was small and nonsignificant (mean difference = 0.1409 μV, adjusted *p* = 0.6958, Cohen’s d = 0.1558). These results describe limited LPP separation between medium and high friendliness ratings, without establishing a physiological threshold or equivalence.

**Table 5:** Tukey *post hoc* contrasts across rating categories.

Feature	Contrast	Mean diff.	SEdiff	*q*	*p* adj.	95% CI	Cohen’s d
Pupil (mm)	Low vs. Medium	0.3372	0.0620	7.6892	< 0.001	[0.1903, 0.4840]	1.0670
Pupil (mm)	Low vs. High	0.6993	0.0688	14.3703	< 0.001	[0.5363, 0.8622]	2.1030
Pupil (mm)	Medium vs. High	0.3621	0.0638	8.0317	< 0.001	[0.2112, 0.5131]	1.1681
FAA	Low vs. Medium	0.1133	0.0220	7.2672	< 0.001	[0.0611, 0.1654]	1.0518
FAA	Low vs. High	0.2401	0.0231	14.6758	< 0.001	[0.1854, 0.2949]	2.3075
FAA	Medium vs. High	0.1269	0.0206	8.7056	< 0.001	[0.0781, 0.1757]	1.1445
SCR (μS)	Low vs. Medium	0.3666	0.0463	11.1958	< 0.001	[0.2569, 0.4762]	1.7281
SCR (μS)	Low vs. High	0.7834	0.0494	22.4053	< 0.001	[0.6663, 0.9005]	3.3690
SCR (μS)	Medium vs. High	0.4168	0.0451	13.0749	< 0.001	[0.3101, 0.5236]	1.6778
LPP (μV)	Low vs. Medium	0.6925	0.1781	5.5000	< 0.001	[0.2709, 1.1141]	0.7466
LPP (μV)	Low vs. High	0.8334	0.1902	6.1980	< 0.001	[0.3831, 1.2836]	1.0068
LPP (μV)	Medium vs. High	0.1409	0.1734	1.1492	0.6958	[−0.2696, 0.5514]	0.1558

Because the same participants contributed multiple observations, these contrasts are interpreted as descriptive follow-up comparisons to Table 4 rather than as independent-sample evidence. CI, confidence interval.

Figure 1 visualizes the distributions of pupil diameter, FAA, SCR amplitude, and LPP amplitude across the corresponding low, medium, and high rating categories. The plots reinforce the category-separation patterns summarized numerically in Tables 4, 5, particularly the monotonic shifts in pupil diameter, FAA, and SCR amplitude.

**Figure 1:**
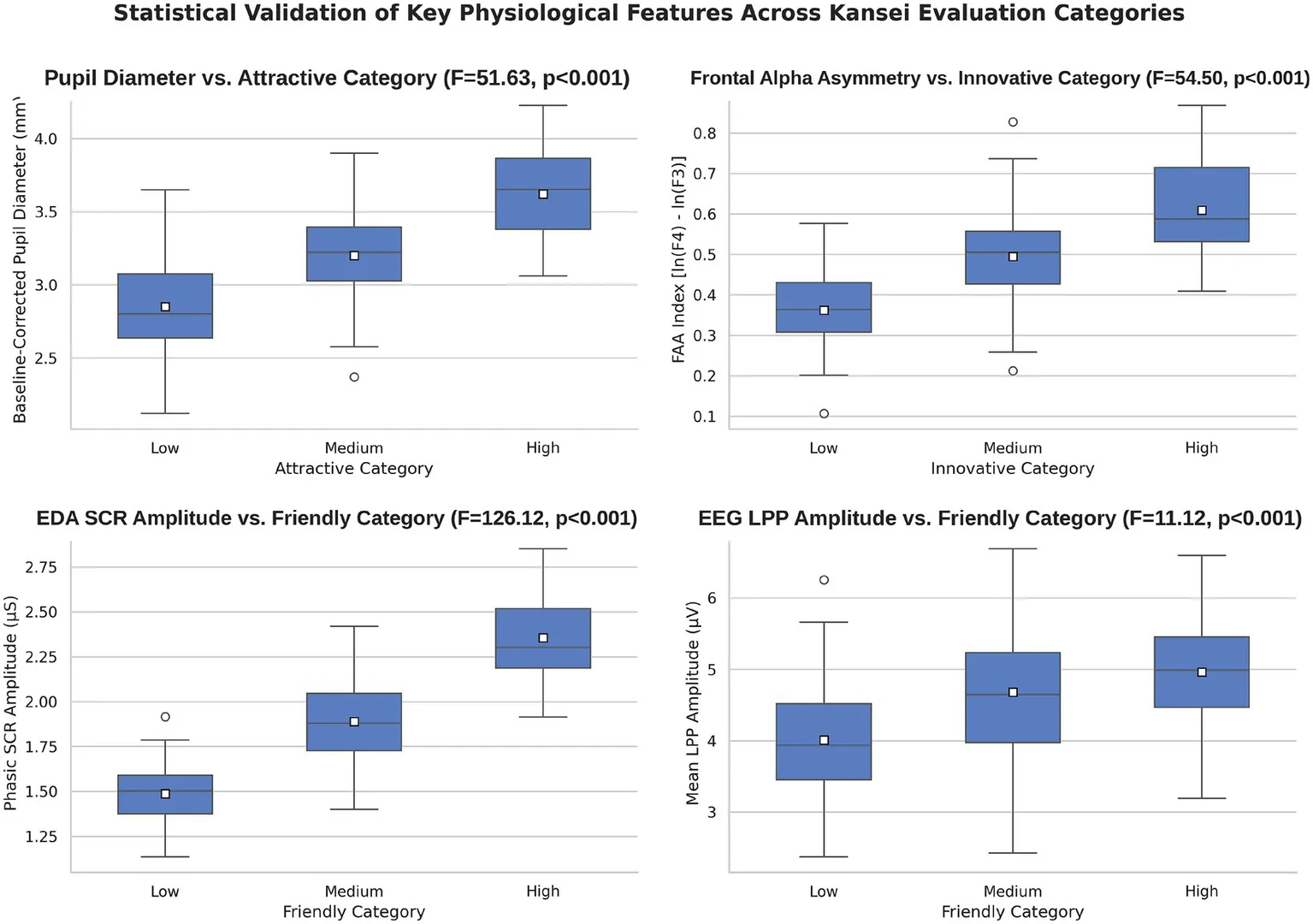
Physiological feature distributions across rating categories. Note. The F statistics shown in this figure correspond to the ANOVA results in Table 4: *F* = 51.63 for pupil diameter, *F* = 54.50 for FAA, *F* = 126.12 for SCR amplitude, and *F* = 11.12 for LPP amplitude.

### 4.3 Predictive performance relative to representative fusion models

Table 6 compares GAMAF with early-fusion SVR, late-fusion MLP, TFN, LMF, and MulT. GAMAF had the highest aggregate *R*^2^ and lowest MAE and RMSE for each target. For attractiveness, *R*^2^ increased from 0.75 with MulT to 0.81 with GAMAF, while MAE decreased from 0.25 to 0.18. Corresponding innovativeness values were 0.78 versus 0.85 for *R*^2^ and 0.22 versus 0.15 for MAE. Friendliness values were 0.76 versus 0.84 for R^2^ and 0.24 versus 0.17 for MAE. Across targets, mean *R*^2^ was 0.833 for GAMAF and 0.763 for MulT, an absolute difference of 0.070. Mean MAE and RMSE were approximately 29.6 and 29.7% lower, respectively. Section 4.6 reports GAMAF fold-level *R*^2^ variability; between-model differences remain descriptive because matched baseline fold statistics and participant-level intervals were not quantified.

**Table 6:** Regression performance across three product-impression targets.

Model	Attractiveness *R*^2^ / MAE / RMSE	Innovativeness *R*^2^ / MAE / RMSE	Friendliness *R*^2^ / MAE / RMSE
Early fusion SVR (RBF)	0.52/0.45/0.58	0.48/0.49/0.63	0.51/0.46/0.59
Late fusion MLP	0.55/0.42/0.54	0.53/0.44/0.56	0.56/0.41/0.53
Tensor fusion network (TFN)	0.68/0.31/0.40	0.70/0.30/0.38	0.67/0.33/0.42
Low-rank multimodal fusion (LMF)	0.71/0.28/0.36	0.73/0.27/0.35	0.70/0.29/0.37
Multimodal transformer (MulT)	0.75/0.25/0.32	0.78/0.22/0.28	0.76/0.24/0.31
GAMAF (Proposed)	0.81/0.18/0.23	0.85/0.15/0.19	0.84/0.17/0.22

MAE and RMSE are expressed in rating units; *R*^2^ is dimensionless and is distinct from classification accuracy. Section 4.6 reports GAMAF fold-level *R*^2^ dispersion. Baseline fold statistics, error-metric dispersion, repeated-seed estimates, and confidence intervals were not quantified, so comparisons are interpreted descriptively. RBF, radial basis function; SVR, support vector regression; MLP, multilayer perceptron; TFN, Tensor Fusion Network; LMF, low-rank multimodal fusion; MulT, Multimodal Transformer.

Performance ordering was consistent across *R*^2^, MAE, and RMSE, indicating that the observed advantage of GAMAF was not limited to a single evaluation criterion. Figure 2 therefore visualizes *R*^2^ for clarity, whereas Table 6 provides the complete set of regression metrics used for model comparison.

**Figure 2:**
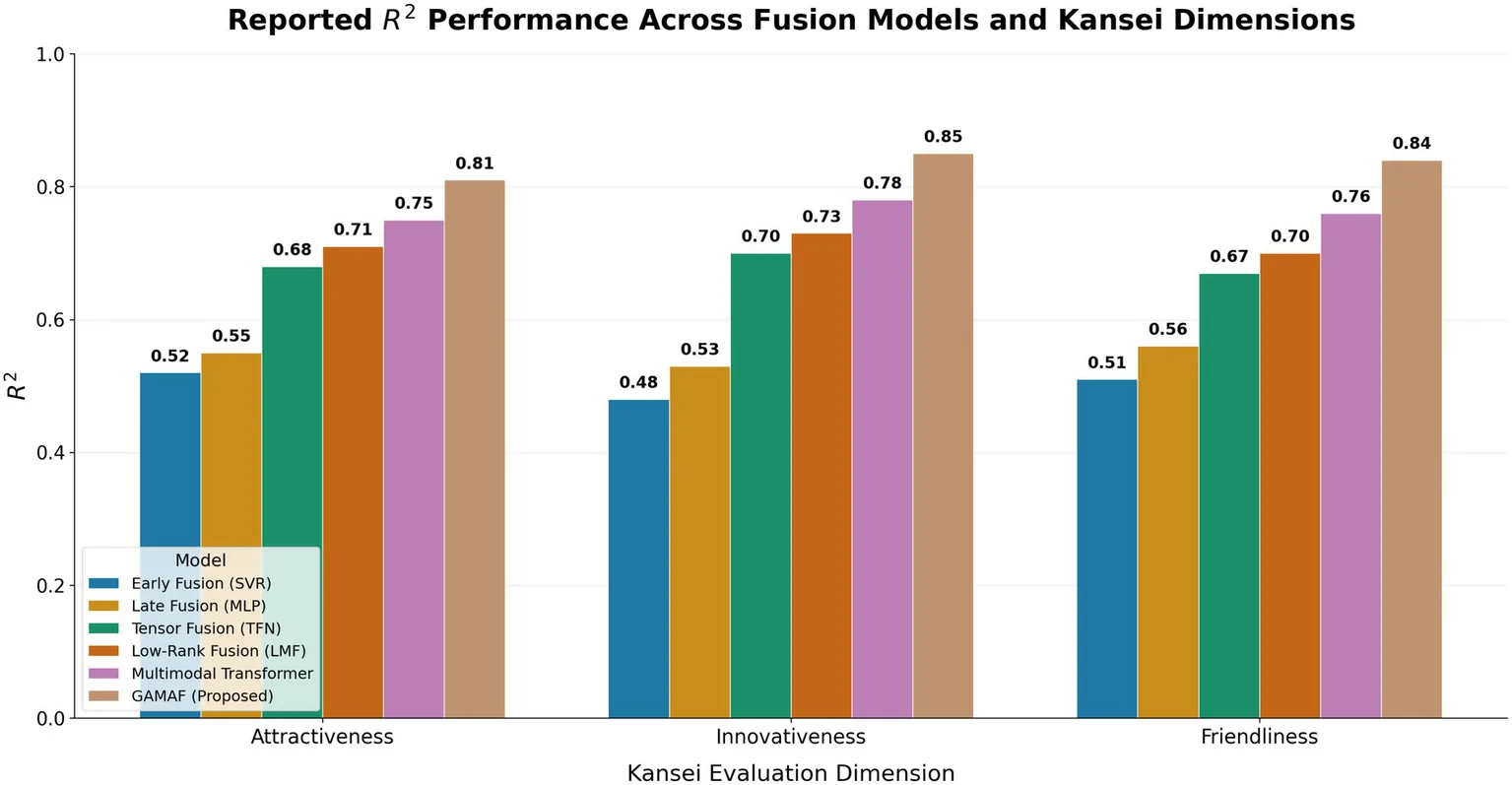
*R*^2^ values by model and rating target. the figure presents *R*^2^ as the vertical-axis performance metric for visual comparison across models and rating targets. Complete *R*^2^, MAE, and RMSE values are provided in Table 6.

### 4.4 Ablation analysis

Table 7 summarizes performance after individual GAMAF components were removed or replaced. Removing generative augmentation reduced mean *R*^2^ from 0.833 to 0.700 (Δ*R*^2^ = −0.133). Removing cross-modal attention yielded mean *R*^2^ = 0.760 (ΔR^2^ = −0.073), while removing graph interaction yielded 0.733 (Δ*R*^2^ = −0.100). Replacing adaptive gating with equal weights produced the largest reduction, to 0.637 (Δ*R*^2^ = −0.197). Replacing gating with concatenation yielded mean *R*^2^ = 0.713 (Δ*R*^2^ = −0.120). Each modification reduced performance across all three targets, supporting evaluation of the integrated configuration rather than attribution to a single component.

**Table 7:** Component ablation analysis.

Variant	Component changed	Attractiveness *R*^2^ / MAE	Innovativeness *R*^2^ / MAE	Friendliness *R*^2^ / MAE
M0 (Full GAMAF)	None (Proposed)	0.81/0.18	0.85/0.15	0.84/0.17
M1 (without Generative)	Generative Augmentation	0.69/0.28	0.71/0.27	0.70/0.29
M2 (without Cross-Attn)	Cross-Modal Attention	0.74/0.23	0.78/0.21	0.76/0.24
M3 (without GNN)	GNN Topology (GAT)	0.72/0.25	0.75/0.23	0.73/0.26
M4 (without Gating - Avg)	Gating (Equal Weights)	0.65/0.31	0.62/0.34	0.64/0.32
M5 (without Gating - Concat)	Gating (Concatenation)	0.70/0.27	0.73/0.24	0.71/0.28

Ablation differences describe changes associated with component removal or replacement. Fold-level dispersion, confidence intervals, and repeated-seed estimates were not quantified for these variants. Differences can reflect representation capacity, optimization, and regularization as well as the function of the modified component.

Replacing adaptive gating with equal weights produced the largest observed reduction, followed by removal of generative augmentation and graph interaction. Removing cross-modal attention produced a smaller reduction, with its contribution dependent on subsequent graph propagation. These results favor target-dependent fusion over fixed equal weighting within the tested configuration but do not establish a unique mechanism for the performance differences.

### 4.5 Modality weights and exploratory visualizations

Mean gate weights differed across targets. ET received the largest weight for attractiveness (63.1%), EEG for innovativeness (72.5%), and peripheral physiology for friendliness (71.3%). The remaining representations received lower weights for each respective target. These coefficients describe model allocation after cross-modal interaction rather than percentages of physiological causation.

Figures 3, 4 show the trial-level gate-weight distributions summarized in Table 8. Attractiveness predictions emphasized ET, innovativeness predictions emphasized EEG, and friendliness predictions emphasized peripheral physiology. Within-target dispersion showed that weighting varied across trials rather than following a single fixed profile.

**Figure 3:**
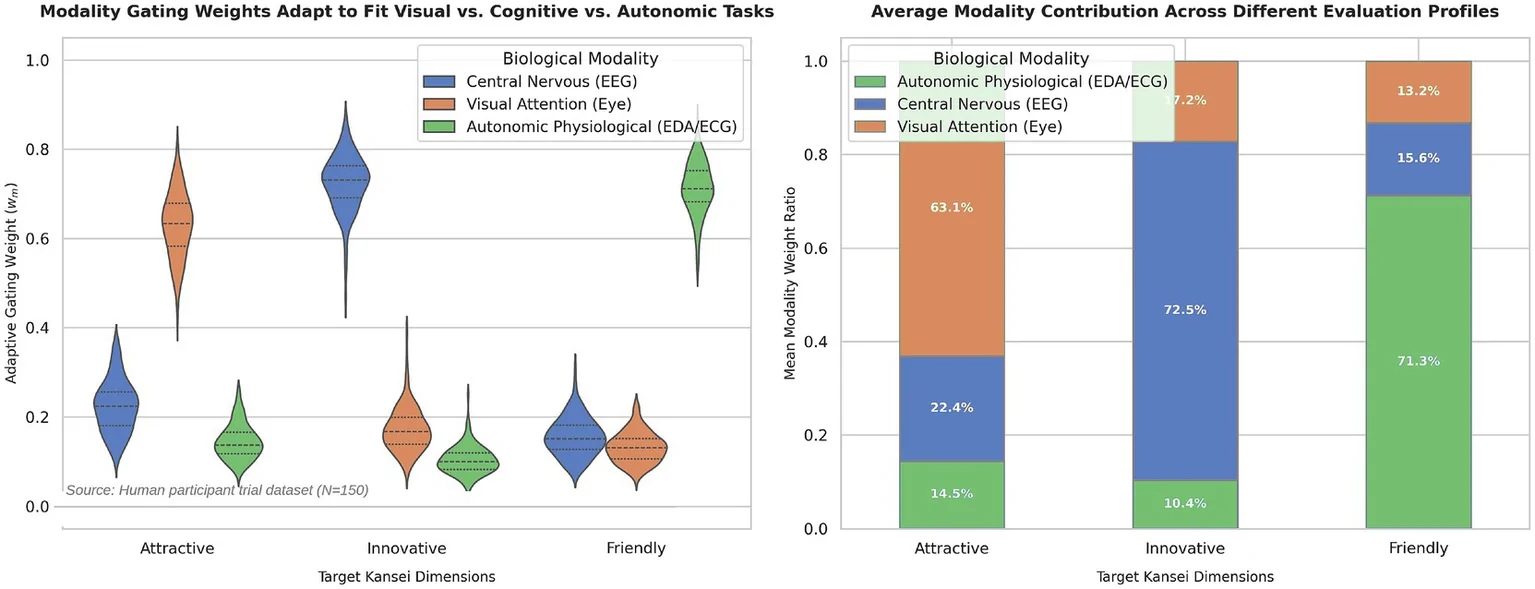
Gate-weight distributions and mean weights. The figure uses consistent modality labels and color mapping across both panels and summarizes gate-weight distributions for the 150 participant–stimulus trials. The displayed values represent learned model weights rather than independent physiological contribution percentages.

**Figure 4:**
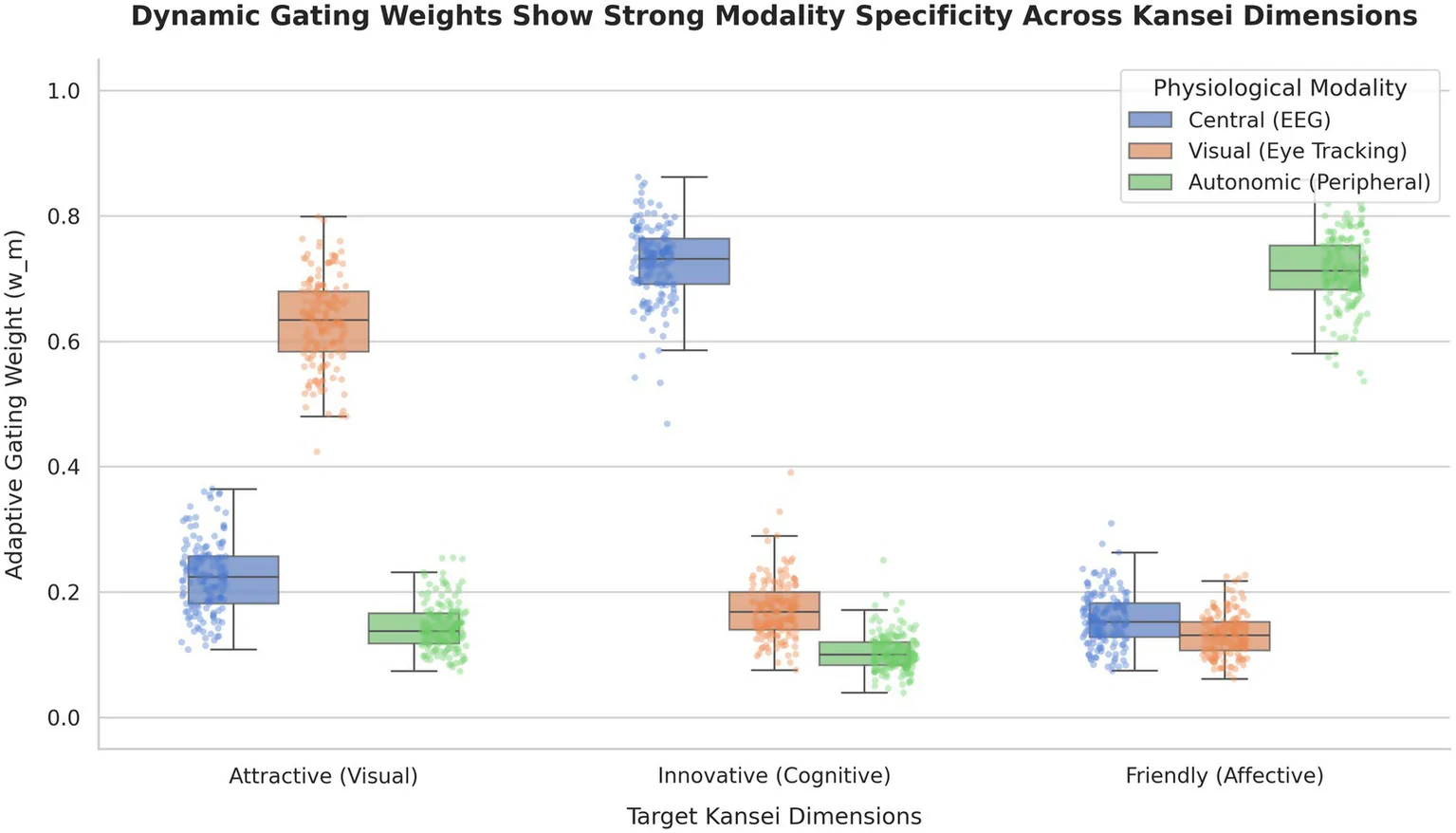
Trial-level variation in modality gate weights. The distributions describe trial-level variability and target-dependent emphasis in adaptive gate outputs. They complement the mean weights in Table 8 and do not constitute independent inferential evidence.

**Table 8:** Mean modality gate weights by product-impression target (%).

Target	EEG	ET	Peripheral
Attractiveness	22.4	63.1	14.5
Innovativeness	72.5	17.2	10.4
Friendliness	15.6	13.2	71.3

The innovativeness row sums to 100.1% because of rounding. Because graph propagation mixes information before gating, these percentages quantify the model’s weighting of modality-labeled latent representations rather than exclusive sensor-level contributions.

Figure 5 shows exploratory correlations between selected physiological features and the three rating targets. Prominent associations included FAA with innovativeness, pupil diameter with attractiveness, and SCR amplitude with friendliness, while most cross-feature correlations were smaller. These patterns complement the gate-weight analysis but do not establish physiological connectivity, causal direction, or unique construct specificity. Repeated observations within participants further limit an independence-based interpretation of the coefficients.

**Figure 5:**
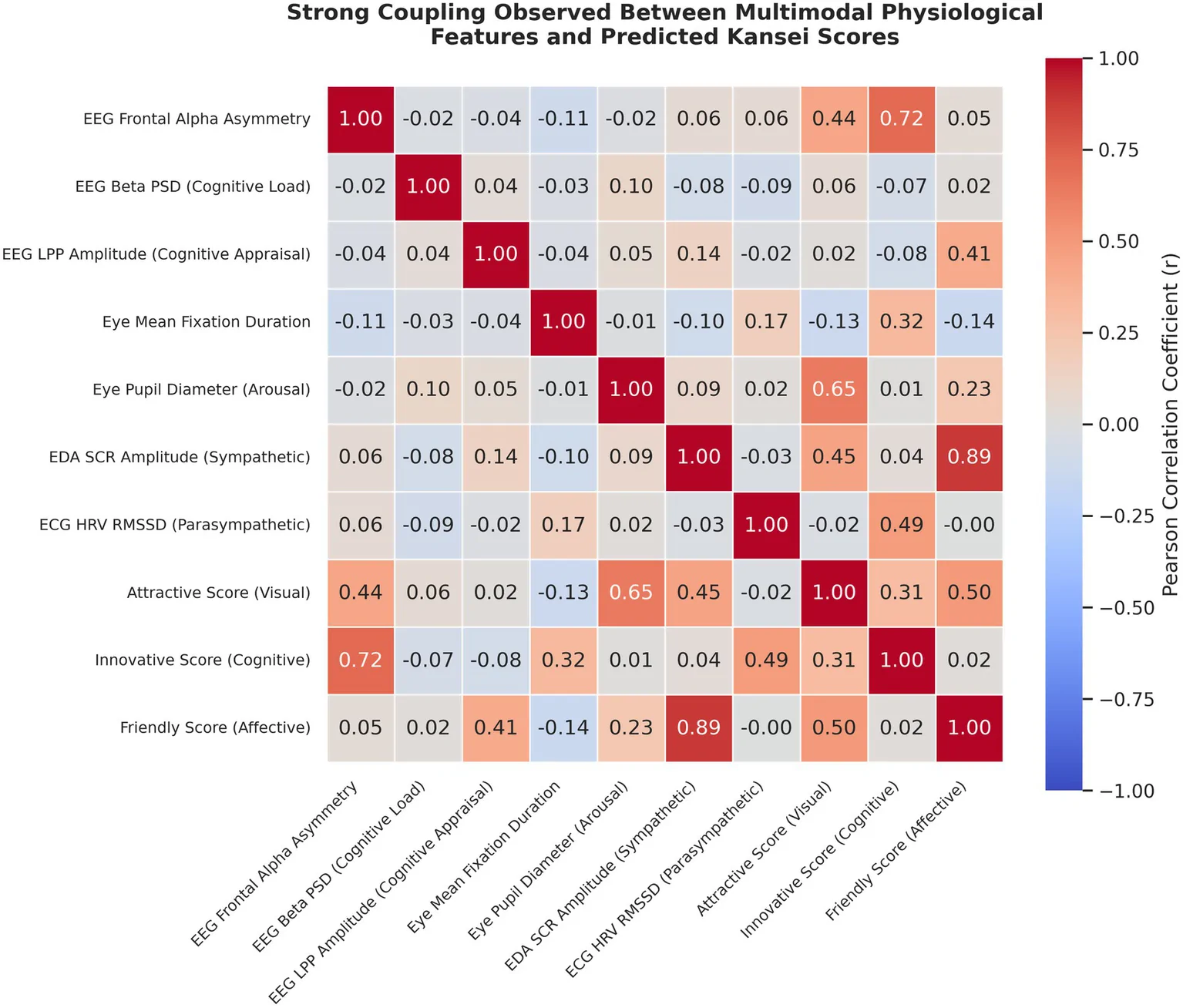
Exploratory correlation matrix of features and rating variables. The figure is an exploratory feature–rating correlation display. Correlations quantify pairwise association within the trial-level dataset and are not interpreted as evidence of causal physiological coupling. Because participants contributed repeated trials, the matrix is used to support qualitative interpretation of feature patterns rather than formal population inference.

Figure 6 presents two-dimensional t-distributed stochastic neighbor embedding (t-SNE) projections of the learned representations, grouped by rating category (Van der Maaten and Hinton, 2008). The projections displayed separation among trials with different rating levels. Because t-SNE emphasizes local neighborhood structure, separation does not establish original-space linear separability or generalization to unseen participants and designs. These exploratory visualizations complement the held-out regression results without providing an independent predictive-performance estimate.

**Figure 6:**
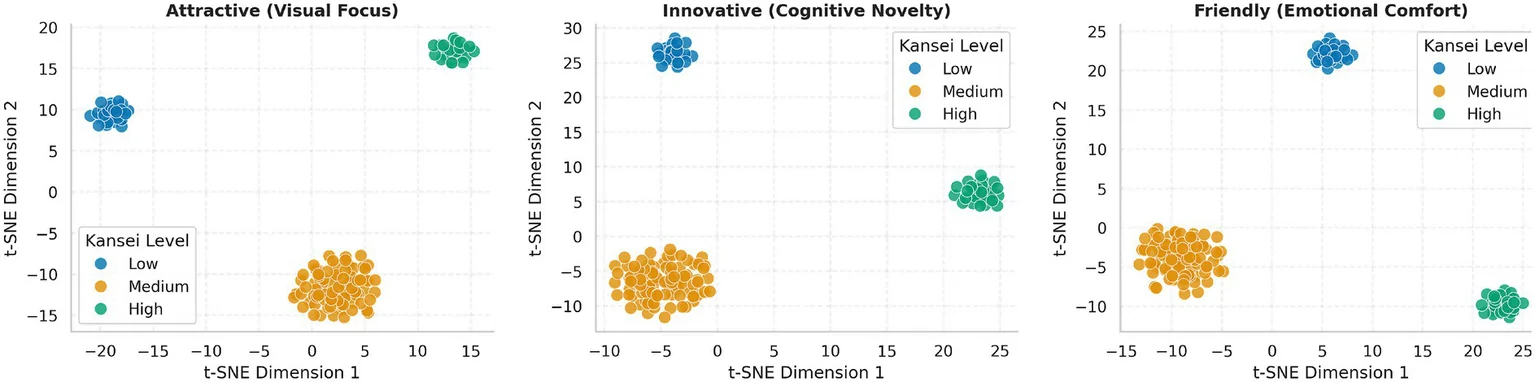
Exploratory t-SNE projections grouped by rating category. The figure is used only to visualize the structure of the learned latent representation. Apparent cluster separation should not be interpreted as an independent measure of classification accuracy or out-of-sample generalization; predictive claims are based on the held-out regression results in Table 6.

### 4.6 Outer-fold performance variability and computation time

Table 9 reports GAMAF R^2^ values for the five outer folds. Arithmetic means were 0.810 for attractiveness, 0.850 for innovativeness, and 0.840 for friendliness. Each target had sample SD = 0.0158, calculated from the five displayed values using K − 1 = 4 as the denominator. The folds share training observations and therefore do not represent five independent experimental replications. These summaries describe split-dependent variability; fold-level error dispersion, baseline variability, and repeated-seed uncertainty were not quantified.

**Table 9:** GAMAF outer-fold R^2^ values and computational measurements.

Fold	Attractiveness *R*^2^	Innovativeness *R*^2^	Friendliness *R*^2^	Training time (s)	Inference (ms)
1	0.82	0.86	0.83	14.2	2.4
2	0.79	0.84	0.85	13.8	2.4
3	0.83	0.87	0.86	14.5	2.4
4	0.80	0.83	0.82	13.9	2.4
5	0.81	0.85	0.84	14.1	2.4
Mean	0.810	0.850	0.840	14.10	2.4
SD	0.016	0.016	0.016	0.27	—

Fold summaries use arithmetic means and sample standard deviations; *R*^2^ is dimensionless. Inference measurements concern prediction from a feature vector rather than the six-second viewing interval or the complete design-feedback workflow. The dash indicates that inference-latency SD is not reported. No stage-resolved or matched baseline efficiency comparison is established by these measurements.

Computational measurements used a workstation equipped with an NVIDIA GeForce RTX 4090 GPU with 24 GB of memory. Recorded training times averaged 14.10 s per fold (sample SD = 0.27 s), with a five-fold sum of 70.5 s. Inference latency was 2.4 ms per feature vector in each fold. Identical fold values at this precision yield zero observed between-fold dispersion and do not quantify within-fold timing variability. These measurements describe the recorded runs rather than establish complete pipeline cost or relative efficiency across models.

## 5 Discussion

### 5.1 Contribution to affective product evaluation

GAMAF integrates neural, visual-attentional, and autonomic information to predict product impressions. The full configuration achieved higher aggregate R^2^ and lower errors than the evaluated early, late, tensor, low-rank, and transformer fusion baselines. Relative to MulT, mean R^2^ increased by 0.070 and mean MAE and RMSE decreased by approximately 30%. Component removal or replacement reduced explained variance, supporting the integration of augmentation, cross-modal interaction, and adaptive weighting within this task. GAMAF outer-fold R^2^ standard deviations were approximately 0.016 for each target. This limited split-dependent dispersion does not establish repeated-training stability or statistical significance of the differences between models.

The framework extends computational Kansei engineering by linking explicit product-impression ratings to concurrent physiological responses. Earlier work mapped subjective impressions to product attributes, while recent approaches incorporate generative design and physiological measurement (Schütte et al., 2004; Yang et al., 2023; Zhu et al., 2025). GAMAF provides a predictive representation layer that preserves self-reports as learning targets and summarizes target-dependent use of multimodal information. This connection may support concept screening and selection of designs for more detailed user evaluation.

Prediction of subjective ratings establishes correspondence with those ratings rather than evidence that physiology constitutes a more objective ground truth. Physiological responses can complement or diverge from self-report, but interpreting such divergence requires an independent behavioral or experiential criterion (Mauss and Robinson, 2009). Relevant criteria include choice, repeated preference, task performance, and long-term use. Multimodal predictions should therefore supplement users’ expressed preferences when informing design decisions.

Kansei-oriented product prediction differs from conventional emotion recognition in both its targets and evaluation context. GAMAF estimates design-related impressions from numerically coded five-point ratings rather than assigning trials to emotion categories. DEAP and DREAMER also contain affective-dimension ratings that can support regression, so public affective datasets are not exclusively categorical (Katsigiannis and Ramzan, 2018; Koelstra et al., 2012). The distinction instead concerns product-specific judgments, stimuli, and design decisions. Emotion-classification accuracy and the R^2^ or rating-scale errors reported here address different tasks and cannot be compared directly.

### 5.2 What multimodal integration can and cannot explain

The modalities provide complementary information, but their psychological interpretations overlap. Pupil changes reflect luminance, effort, and autonomic activation, while EEG features can reflect attention, appraisal, and artifacts. SCR amplitude indicates sympathetic activation without uniquely identifying positive or negative experience (Benedek and Kaernbach, 2010; Mathôt, 2018; Smith et al., 2017). Prediction can exploit these associations without identifying a unique underlying process. For example, interface complexity could affect both gaze and innovativeness ratings without implying an innovation-specific neural representation.

The graph represents computational interactions rather than biological signal flow. Three modality-level nodes based on trial summaries cannot resolve the temporal ordering of cortical, visual, and autonomic events. Testing physiological pathways would require time-resolved measurements and models comparing alternative directions of influence. The present results instead associate learned cross-modal interactions with improved prediction within the tested architecture.

Gate weights provide comparative diagnostics of target-dependent model behavior. Their values depend on representation scale, cross-modal mixing, correlated inputs, and optimization. Attention-like coefficients do not necessarily provide complete explanations of neural predictions (Jain and Wallace, 2019; Serrano and Smith, 2019). Table 8 therefore supports hypotheses about the use of modality-labeled representations rather than exclusive sensor-level contributions.

The target-dependent patterns suggest testable information-processing hypotheses. A larger ET weight for attractiveness may reflect visual exploration and attention during appraisal of cockpit composition. Pupil variation may also reflect luminance or effort rather than esthetic pleasure (Mathôt, 2018). Greater EEG emphasis for innovativeness could reflect processing of unfamiliar layouts, but novelty was not isolated from complexity, attention, or motivational engagement. FAA is not an innovation-specific marker (Smith et al., 2017). Greater peripheral weighting for friendliness may reflect autonomic engagement during approachability judgments, without uniquely identifying comfort, trust, or positive affect (Benedek and Kaernbach, 2010). Cross-modal mixing, correlated predictors, and design properties remain alternative explanations requiring controlled evaluation beyond the present stimuli.

### 5.3 Augmentation as a conditional modeling assumption

The augmentation benefit is conditional on the model and task rather than evidence that synthetic features are physiologically equivalent to real observations. MMD and Wasserstein distance describe selected aspects of distributional agreement, while downstream performance measures predictive usefulness. Neither alone establishes preservation of the biological data-generating process. Feature-space cWGAN-GP augmentation is therefore interpreted as a training-distribution enrichment strategy under limited participant data.

The non-monotonic pattern in Table 3 is particularly informative. Increasing augmentation from 50 to 200% improved both distributional similarity and auxiliary performance, whereas further increases to 300 and 400% degraded both measures. This pattern is compatible with a trade-off between beneficial coverage of the training manifold and excessive influence of generated observations. It also indicates that augmentation ratio should be treated as a task-specific hyperparameter rather than as a universal rule. The 200% ratio is therefore an empirical choice for this dataset, not a general recommendation for multimodal psychophysiological modeling.

Augmentation assessment should distinguish distributional agreement, memorization, and downstream utility. Nearest-neighbor comparisons can assess copying, while conditional feature summaries can examine preservation of label-dependent structure. Held-out real-data performance provides the direct test of predictive usefulness. Any diagnostic influencing model selection belongs within the training and validation partitions (Kapoor and Narayanan, 2023; Kapoor et al., 2024; Rosenblatt et al., 2024).

### 5.4 Generalization, statistical dependence, and practical use

The study includes 50 independent participants but only three shared cockpit stimuli. Participant-wise validation estimates prediction for new participants viewing this stimulus set; it does not establish transfer to unseen cockpit designs. Deployment-oriented evaluation requires more diverse stimuli and design-held-out testing, ideally combined with participant-held-out validation. A model can generalize across individuals while relying on design-specific patterns that fail to transfer to new product forms.

Related EEG research has explicitly addressed cross-subject and cross-dataset distribution shifts through graph-based adversarial learning and domain adaptation (Chen et al., 2024; Imtiaz and Khan, 2025). These studies are relevant because they treat generalization as a distribution-shift problem rather than merely increasing model capacity. Similar strategies could be incorporated into GAMAF in future work, particularly when extending the framework to different product categories, acquisition systems, or populations. Such extensions would provide a stronger test of whether the learned multimodal representation captures transferable affective information.

Repeated observations also constrain physiological inference. Trial-level associations can reflect participant baselines, stimulus properties, or their interaction. Mixed-effects models with participant random intercepts and fixed stimulus effects can account for part of this dependence (Bates et al., 2015). Participant-level and, with sufficient stimulus coverage, design-level uncertainty would align inferential claims with the intended populations of users and products.

In design practice, predicted Kansei ratings could support early-stage screening of concepts viewed by users during physiological acquisition. Combined with explicit ratings and qualitative feedback, these estimates could guide concept prioritization and reveal trade-offs among attractiveness, innovativeness, and friendliness. GAMAF requires responses elicited by viewing a candidate; it does not score product images directly. After uncertainty calibration, adaptive evaluation could allocate additional testing to candidates with uncertain or conflicting assessments. Personalized optimization would require individual calibration, repeated within-user validation, and transfer to new designs. Neither personalization nor reduced testing burden was demonstrated here. Deployment also requires robustness to sensor dropout, lower calibration burden, and sufficient prediction accuracy to distinguish consequential design alternatives.

### 5.5 Conceptual integration with generative product design

A future human-in-the-loop workflow could connect GAMAF to a generative design model through iterative user evaluation. A conditional generator could produce cockpit candidates subject to geometric, functional, and visual constraints. Users would view these candidates during multimodal recording, and GAMAF would estimate their attractiveness, innovativeness, and friendliness ratings. Designers could retain the scores as separate objectives or combine them using explicit preference weights. Candidate rankings could then guide selection or conditioning in the next generation round. A design-conditioned reward model offers a further extension but requires a diverse evaluated design set and independent validation.

This workflow would use inferred product impressions to guide evaluation and selection rather than directly update a generator from physiological signals. Human review would retain control over feasibility, usability, and design objectives, while fresh user evaluations would test whether proposed changes improve the intended impressions. Uncertainty calibration and design-held-out validation would help identify optimization toward prediction artifacts. The present study evaluates the decoding stage only and does not demonstrate generative iteration, real-time neurofeedback, or closed-loop improvement. The proposed integration extends Kansei-guided generative design conceptually (Yang et al., 2023) and requires prospective evaluation of the complete workflow.

### 5.6 Computational requirements and deployment feasibility

GAMAF requires additional training stages beyond a single fusion predictor. Computational cost includes generator fitting, feature generation and selection, supervised representation learning, and participant-grouped model selection. At 200% augmentation, predictor fitting uses three times the corresponding number of real-only feature vectors. This sample increase does not imply proportional runtime because batching, optimization, and hardware affect computational cost. The generative stage is confined to training; inference uses fitted preprocessing and the fusion predictor without synthesizing additional observations.

The fusion stage comprises three 64-dimensional representations, four-head attention, a three-node graph, and a 32-unit regression hidden layer. The small graph limits modality interactions, but attention and propagation add operations relative to simpler fusion methods. Unlike early-fusion SVR and late-fusion MLP, GAMAF also requires adversarial training. Relative cost against TFN, LMF, and MulT depends on implementation, dimensions, and training configuration; predictive improvement does not establish computational advantage. Section 4.6 reports descriptive timing measurements without a matched comparison of full-pipeline cost, memory, or energy use. Predictor latency alone does not represent complete workflow latency, which also includes viewing, acquisition, preprocessing, design generation, and human review. Practical benchmarking should distinguish final predictor fitting from generator training, feature selection, and hyperparameter search under matched hardware and validation splits.

### 5.7 Specific limitations and validation priorities

Several limitations constrain interpretation. The participant sample comprises 50 individuals, and only three cockpit designs represent the product space. The 150 retained trials are repeated observations within these individuals and shared stimuli rather than 150 independent participants or products. GAMAF fold-level R^2^ summaries quantify split-dependent dispersion but not baseline or ablation variability, error-metric uncertainty, repeated-seed effects, or participant-level confidence intervals. Independent replication across broader participant and design samples is needed to assess performance stability and comparative superiority.

Measurement and construct validity also limit interpretation. Spectral HRV indices were excluded because the six-second interval cannot support conventional frequency-domain estimation. Retained RMSSD and SDNN estimates remain sensitive to valid-beat counts and artifact correction and are not interchangeable with conventional longer-window measures. Free viewing complicates interpretation of stimulus-locked ERP components. The five-point ratings are ordinal but were treated as approximately interval-scaled in regression. Ordinal-model sensitivity analyses and task-specific reliability assessments would strengthen the measurement basis for design applications.

Architectural diagnostics also have limited explanatory scope. Attention coefficients and gate weights characterize model behavior without uniquely identifying causal physiological mechanisms. The graph represents modality-level features rather than channel-level or time-resolved physiological networks. Temporal encoders, uncertainty-aware gating, and external behavioral criteria could test whether learned interactions transfer across participants, tasks, and product categories. Such validation would clarify the relationship between predictive fusion and physiological processes underlying product experience.

## 6 Conclusion

GAMAF combines feature-space augmentation, cross-modal attention, graph interaction, and adaptive gating to predict product impressions from EEG, ET, and peripheral physiology. Participant-wise cross-validation yielded mean outer-fold *R*^2^ values of 0.810, 0.850, and 0.840 for attractiveness, innovativeness, and friendliness, each with sample SD approximately 0.016. Aggregate comparisons favored GAMAF over the evaluated fusion baselines, while ablations supported the integrated architecture. These findings support multimodal psychophysiology as complementary evidence for Kansei-oriented evaluation within the present cockpit task. Broader participant samples, unseen designs, matched uncertainty estimates, and behavioral validation are needed to establish transferability. Predicted Kansei ratings could guide future generative design iterations, although closed-loop improvement and personalization remain prospective applications.

## Data Availability

The raw data supporting the conclusions of this article will be made available by the authors, without undue reservation.
